# Hyperbranched Polyglycerol Derivatives as Prospective Copper Nanotransporter Candidates

**DOI:** 10.3390/molecules23061281

**Published:** 2018-05-26

**Authors:** Mohiuddin Quadir, Susanne Fehse, Gerhard Multhaup, Rainer Haag

**Affiliations:** 1 Department of Coatings and Polymeric Materials, North Dakota State University, Fargo, ND 58105, USA; 2 Institute of Chemistry and Biochemistry, Freie Universität Berlin, Berlin 14195, Germany; fehse@zedat.fu-berlin.de (S.F.); haag@chemie.fu-berlin.de (R.H.); 3 Department of Pharmacology and Therapeutics, McGill University, Montreal, QC H3A 0G4, Canada; gerhard.multhaup@mcgill.ca

**Keywords:** hyperbranched polyglycerol, nanocarrier, ion transport, Cu-deficiency disorders

## Abstract

Hyperbranched polyglycerol (hPG) has been used as a multivalent scaffold to develop a series of nanocarriers capable of high-affinity encapsulation of copper (Cu). A rationally selected set of Cu-complexing motifs has been conjugated to hPG hydroxyl groups to render the constructs potentially usable as exogenous sources of Cu for addressing different pathological conditions associated with Cu-deficiency. We have utilized a newly discovered route to attach Cu-binding domains exclusively within a hPG core by selective differentiation between the primary and secondary hydroxyl groups of the polyol. These hPG-derivatives were found to form a stable complex with Cu ions depending on the type of immobilized ligands and corresponding degree of functionalization. In addition, these Cu-bearing nano-complexes demonstrated moderately cationic surface charge resulting in adjustable protein-binding characteristics and low cellular toxicity profile. We envision that these Cu-loaded hPG nanocarriers can be used as a stable platform to transport the metal ion across the systemic circulation to supply bioavailable quantity of Cu in disease-afflicted tissues.

## 1. Introduction

Recapitulation of homeostatic level of electrolytes or trace element concentration is one of the mainstays of clinical management for a number of pathological conditions, including but not limited to blood disorders, renal failure, acute poisoning, cancer and skeletal system-related diseases. In biological systems, Cu ions are cofactors of several metalloenzymes and are essential for central nervous system (CNS) development [1,2,3,4]. For example, alterations in Cu balance have been linked, but not causally associated, to changes in senile plaque deposition in Alzheimer’s disease (AD) [5]. Currently in clinical trials, exogenous supply of Cu is mediated by soluble oral administration of soluble metal salts or metal complexes in disease conditions where Cu supplementation is required. For example, potential beneficial effects of oral intake of Cu (II)-orotate-dihydrate (8 mg Cu daily) were investigated in AD patients. Although results demonstrated that oral Cu intake has neither a detrimental nor a promoting effect on the progression of AD, Cu treatment stabilized plasma and CSF levels in AD patients and antagonized the unbalanced Cu levels likely by activating the homeostatic system [6,7]. Studies with the antibiotic clioquinol (CQ), an 8-OH quinoline with a moderate affinity for Cu^2+^ and Zn^2+^, the lipophilic chelator (DP109), isatin-Schiff base Cu (II)-complexes and metal bis(thiosemicarbazonato) complexes also confirmed that promoting Cu uptake is a reasonable treatment strategy [8,9,10,11] in Cu deficiency-related disorders. To offset the obvious pharmacokinetic challenges involved with these monovalent, small molecular Cu complexing agents pertaining to systemic toxicity due to complex instability, bioavailability, circulation lifetime and dosing frequency, we have reported a library of macromolecular, multivalent, Cu-encapsulating nanocarrier platforms based on hyperbranched polyglycerol (hPG) [12,13,14]. Due to the presence of multiple chemically accessible hydroxyl groups and very stable ether backbone, as well as high compatibility, we found hPG as an ideal candidate to design Cu complexing systems for therapeutic use. Previously, we showed that the presence of multivalent Cu-complexing ligands on a hPG platform not only increased the affinity and stability of the ligands towards the metal ion, but also suppressed the risk of non-specific toxicity due to premature leaching of the metal ion from the complex. In addition, the synthesized hPG-derived Cu-nanocarriers showed permeation through human brain microvascular endothelial cell (HBMEC) model, which was established to investigate the engagement of these nanocarriers through the blood brain barrier (BBB). It was shown that, hPG-based nanocarriers crossed the BBB model two times more effectively than ^14^C-sucrose and sodium fluorescein (NaFl) and up to 60× better than Evans Blue labeled albumin (EBA), when the the permeability × surface area product (PSe) of the nanocarrier and reference substances were compared [13]. As a polyether, hPG contains a large number of C-O bonds and hydroxyl groups, which makes the molecule highly hydrophilic. In addition, these polyether-based multi-functional scaffolds exhibited condensed three-dimensional structure with a hydrodynamic diameter typically within the range of 3–5 nm, high Cu-loading capacity, intracellular transport and inertness to ubiquitous biological components such as protein and phospholipid membranes. In this study, we elaborate the potential routes to generate hPG-scaffolds, which either complex Cu within a specific molecular domain, or, are decorated with ligands that can either impart superior biocompatibility and putative receptor engagement. The designed architectures were tested for formation of the metal-complex and thermodynamics of the complex stabilization. In addition, we have also investigated electrostatic and surface properties of these dendritic nano-constructs, and evaluated their cellular toxicity and uptake behavior in mammalian cell lines.

## 2. Results

**Scheme 1:**
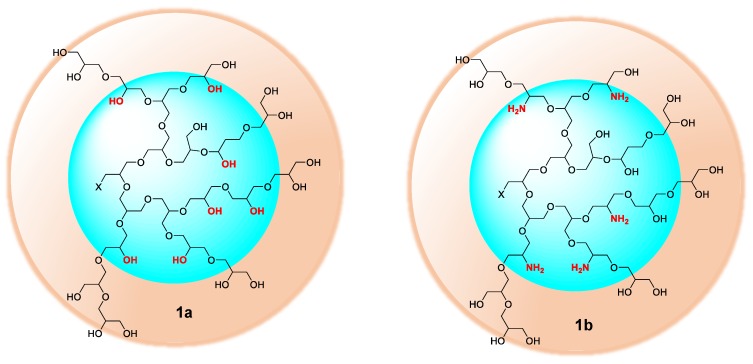
Representative illustration of interior and exterior of hPG (**1a**) scaffold has been indicated by the blue and brown shaded spheres respectively. In structure (**1b**), the interior hydroxyl groups have been converted to amines.

Compared to linear polymers of analogous molecular weight, spatially globular architecture of hPGs gives rise to a number of interesting properties such as reduced viscosities and enhanced water solubility, both of which can be readily modulated by post-synthetic functionalization of peripheral hydroxyl functional group through classical alcohol-group chemistry [15,16,17,18,19,20,21]. Another salient feature that makes hPGs unique from its linear analogues is the fact that these macromolecules exhibit a distinct, convertible ‘interior’ supramolecular space [22], sterically shielded from the topological ‘exterior’ of the scaffold (Scheme 1) [23].

We set out to modify the hPG core structure by orthogonally including/attaching: (a) a Cu encapsulating domain at a specific location within the molecule that can potentially complex Cu ion with substantial stability; and (b) a molecular species that can putatively suppress cytotoxicity and mediate cellular entry. Cu complexing nanocarriers designed in this study are presented in Table 1. We optimized the synthetic design of these hPG-derived metal ion nanocarriers and investigated their capacity to interact with Cu-ion in physiological settings.

### 2.1. Dialkylamine Modified hPG Synthesized through Nucleophilic Substitution Reaction

**Scheme 2:**
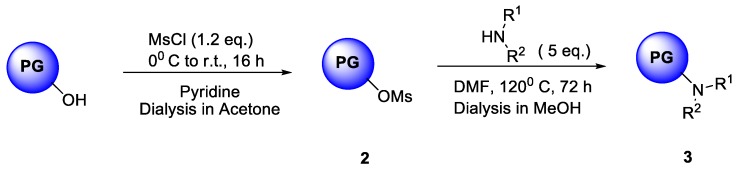
Synthesis of dialkylamine functionalized PG. R^1^ and R^2^ can be similar or different.

As a first approach, a reactive hPG was functionalized with different dialkyl amines (Scheme 2) to obtain a core-shell architecture where the tertiary nitrogen atom bearing alkyl chains acts as the Cu ion complexing modality within the molecule. Water-solubility of these architectures is critically governed by the degree of functionalization of the PG core. Synthetically, these systems were accessible in a straightforward two-step reaction protocol where both terminal (T) and linear (L) hydroxyl group of hPG can be converted to alkyl amine functionality (Scheme 2). Through the formation of an *O*-mesylpolyglycerol [15], compounds 3a–3e can be obtained with quantitative conversion at elevated temperature. 

The critical consideration in this reaction is the high volatility of dialkylamines and the requirement of relatively high temperature (over 100 °C) to carry out the reaction. We have synthesized a series of dialkylamine-substituted hPG following this reaction protocol with satisfactory yield as presented in Table 2.

**Figure 1:**
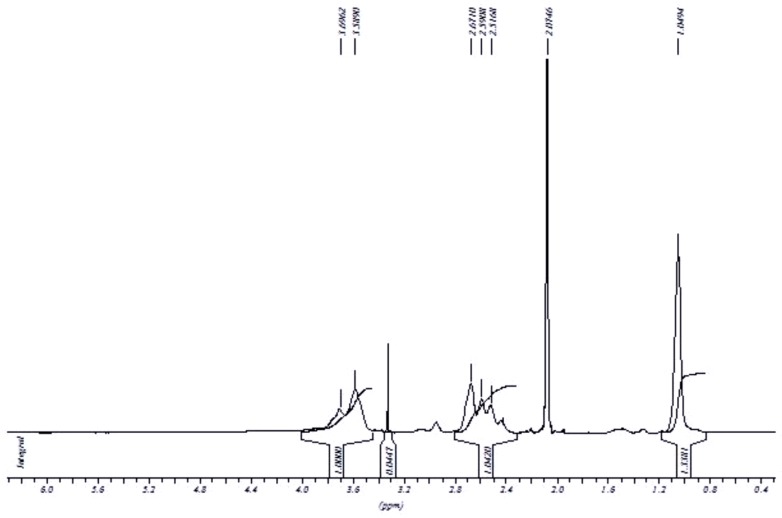
^1^H-NMR of diethylamine functionalized PG (3b) at quantitative functionalization level.

Figure 1 shows the ^1^H-NMR spectrum of **3b**. The broadened signals of the protons of CH_3_ at 1.31 ppm and CH_2_ group from 2.32–2.81 ppm indicate the immobilization of dialkylamine moiety onto the PG scaffold. The degree of functionalization can be estimated from relative integration intensities of the signals from CH_3_ group and that from PG scaffold (3.5–4.0 ppm). In the case of compounds **3c** to **3e**, the degree of functionalization could be easily determined by considering the terminal methyl proton as a reference for calculation. A full synthesis, chemical and biochemical characterization of compound **3f** has been published earlier [13]. Hence, limited experimental data has been presented for this compound for comparison purpose, while, synthetic, UV-spectroscopic, calorimetric and cell cytotoxicity experiment has been conducted and presented for other Cu-nanocarrier candidates.

This is noteworthy that since the signals due to the protons belonging to the alkyl chain of dialkylamines, which are located next to nitrogen atom, overlap with PG signal, therefore the area corresponding to two protons should be subtracted from the PG signal integration to avoid the underestimation of the actual degree of functionalization. The absence of a signal at 3.12 ppm indicates the complete removal of the mesyl group from the PG backbone. Similar ^1^H-NMR patterns were observed with other secondary dialkylamines attached to PG. ^13^C-NMR and IR spectra were also found to be confirmatory for the proposed product structures. 

### 2.2. Selective Chemical Differentiation of Primary and Secondary Hydroxyl Groups of hPG: Cu-Binding Domains at hPG Core

**Scheme 3:**
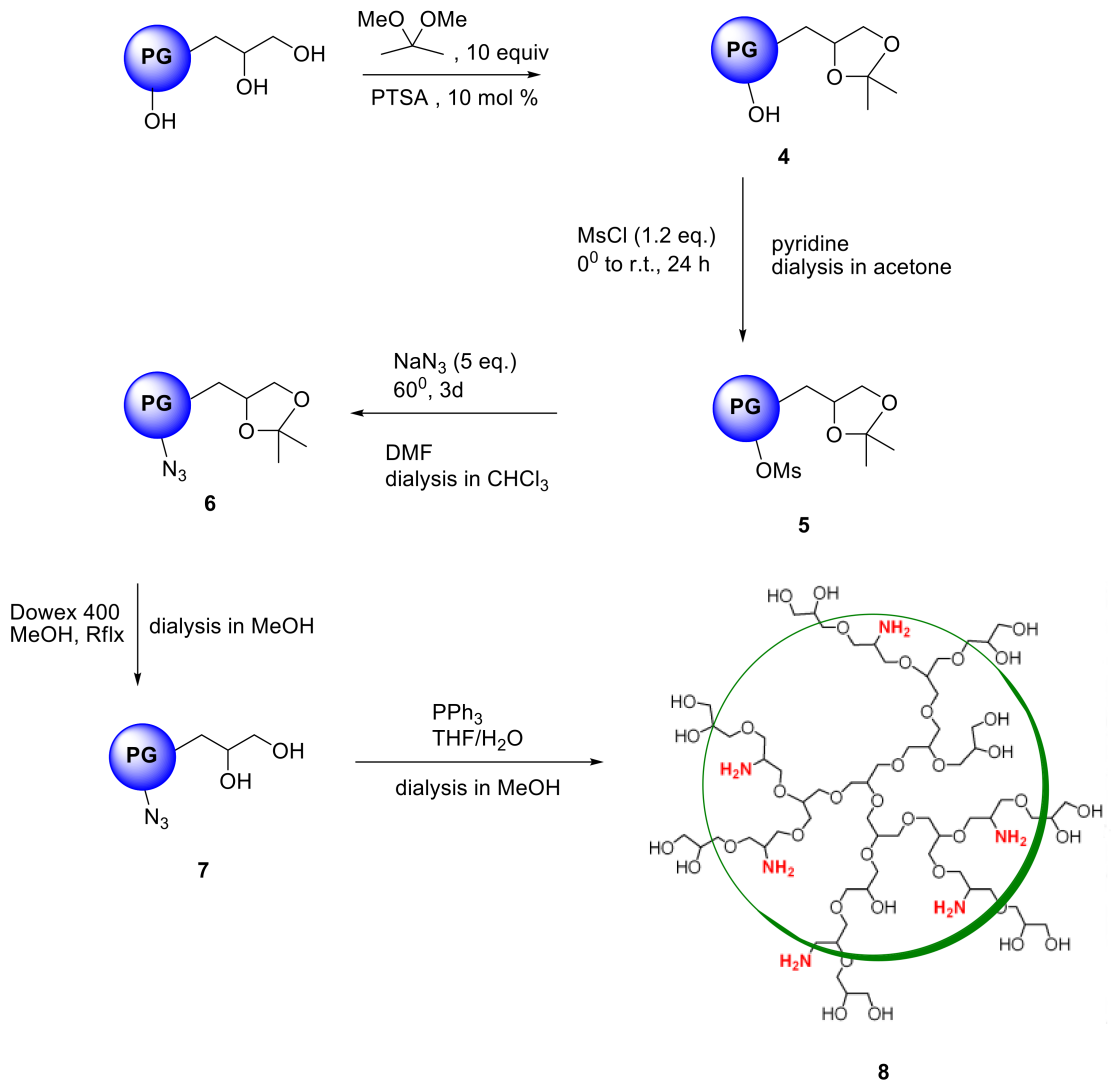
Synthesis of core-functionalized polyglycerolamine **8**.

In contrast to dendrimers, hyperbranched polymers showed no distinguishable interior and periphery. Hyperbranched PGs, however, possesses two types of hydroxyl functional groups (generated from linear and terminal glycerol units), which can be chemically differentiated [24]. Although the linear units are randomly dispersed throughout the structure, they are predominantly present in the proximity of the focal or “core” unit of the macromolecule, when the polymers are prepared by slow monomer addition technique [25]. By following an earlier procedure developed by Haag et al. [22], a chemical differentiation strategy has been undertaken to synthesize core-aminated PG where the “focal-close” linear hydroxyl groups were selectively converted to amines keeping the terminal hydroxyl groups intact. These structural variants of PG were envisioned to encapsulate Cu ion through the nitrogen atoms present in the core, thereby shielding the encapsulation zone. With such structures, it was also possible to retain the required water solubility and biocompatibility profile of the complexes, which are mostly contributed through the “focal-distant” terminal hydroxyl groups. These hyperbranched scaffolds could be envisioned as the molecular chaperons observed in biological systems where the guest molecule is shielded from external environment. The outer hydroxyl group can further be functionalized with targeting moieties or biocompatible PEG shells. In the first step to construct this type of architecture, the terminal 1,2-diols of PG were regioselectively converted to polyacetal **4**. The complete conversion of 1,2-diols in PG was observed (according to ^13^C- and ^1^H-NMR) leaving approximately 40% of the hydroxyl groups unaffected [22]. The synthetic scheme of core-functionalized polyglycerolamine is presented in Scheme 3.

The conversion of **5** from **4** could be monitored by means of IR, by tracking the appearance of azide signal (at 2100 cm^−1^) in polyglycerol azide, **6** [15]. Deprotection of acetals was carried out in the following step with acidic Dowex-400 resin, where the selective acid-catalyzed cleavage of the acetal groups proceeded with quantitative conversion yielding **7**. Core functionalized polyglycerolazide **7** synthesized in this way, where the azide groups are located only at the core of the hyperbranched molecule could also be envisioned as an useful substrate for metal assisted 1,3-Huisgen type “click” chemistry approach. Staudinger reduction of core-functionalized polyglycerolazide carried out in the presence of triphenylphosphine and water, yielded core-functionalized polyglycerolamine (**8**), as confirmed by complete elimination of azide signal in IR spectra. Usually Staudinger reaction is carried out in THF in the presence of small amount of water (5%). However, this is to note that, careful consideration is needed to maintain the ratio of THF to water during the course of the reaction, so that premature precipitation of compound **7** can be optimally minimized. 

### 2.3. Amide Coupling for the Synthesis of hPG-Bispicolylamide Derivatives

Cu^2+^ complexation of a bispicolylamide entity is a widely studied phenomena in designing linker molecules for solid phase peptide synthesis [26]. In peptide chemistry, this type of complexation leads to a weakening of the N-C-amide bond, which can subsequently be cleaved under very mild condition by methanolysis [27,28,29]. In nature, metalloproteases use the same mechanism for hydrolytic cleavage of the peptide bond. Due to high affinity complexation capacity of bispicolylamide derivatives towards Cu, we hypothesized that, bispicolylamide derivatives bound to PG by a stable amide linkage will encapsulate Cu^2+^ ion in aqueous environment with substantial stability. Therefore two carboxylic acid terminated bispicolylamide derivatives, namely NF 135 and KR 455 (a kind gift from AG Bannawarth, Universität Freiburg, Freiburg, Germany) were coupled to core-aminated PG through an amide bond formation to synthesize compounds **11** and **12**, respectively. These derivatives were coupled to core-aminated PG, (**8**, PG_10_-cNH_2_) by EDCI/DMAP mediated coupling protocol. An overnight dialysis in methanol yielded pure compounds **11** and **12** in quantitative yield. ^1^H-NMR conclusively proved the immobilization of the derivatives on to PG scaffold where the broadened signals of protons from the BOC group and those from aromatic pyridine moiety resonate around 1.39 ppm and from 7.0 to 8.57 ppm respectively. Degree of functionalization was limited to approximately 20% in both cases compared to all hydroxyl groups of PG to ensure water-solubility of the products. 

### 2.4. Reductive Amination Pathway towards the Synthesis of Mono-and Oligosaccharide Modified hPG-Amine 

The coupling of mono- and oligosaccharide units to the outer shell of dendritic or hyperbranched architectures has been successful and the resulting amphiphilic macromolecules have been explored as carrier systems for drugs [30,31,32] and DNA [33,34]. These systems, in addition to the increase in biocompatibility, can utilize the molecular recognition potential of mono- and oligosaccharide units for enhanced and selective cell uptake [35,36,37,38,39,40]. PPI dendrimers with a densely organized oligosaccharide shell was proved to be a promising anti-prion agent and extensive studies on oligosaccharide shell coupling to PEI based hyperbranched polymers has been reported [41]. Previously it has been documented that the Cu^2+^ complexes of the polypeptide-shelled PPI dendrimers possess nearly the same stability constants as the Cu^2+^ complexes of the unmodified PPI ones [42]. The study suggested that the polypeptide chains do not alter the Cu^2+^ complexation ability of the dendritic PPI skeleton significantly. Thus, it can be assumed that the dendritic PPI skeleton is the driving force for the complexation of Cu^2+^ and it is independent of the attached peripheral chains as surface groups. In our study, it is therefore presumed that attachment of oligosaccharide shells onto polyglycerolamine will not adversely alter polyglycerolamine’s inherent Cu^2+^ complexation ability. To this end, maltose and *N*-acetylglucosamine have been coupled to polyglycerolamine to utilize the purported recognition potential of carbohydrate motif at glucose transferring receptor (GLUT) present in the BBB. Reductive amination was adopted to immobilize the saccharide units as surface group. This reaction can result in the simultaneous coupling of at least one (or two) saccharide units for one amino surface group with minimized synthetic effort [43]. This synthetic approach has also been described by several authors in immobilizing oligosaccharide onto poly(lysine) and poly- (ornithine) monodendrons and dendrimers, PPI dendrimers [42] and PEI [44] based hyperbranched system. In our case, polyglycerolamine (**13**, fully functionalized, i.e., all hydroxyl groups of PG have been converted to primary amines) was synthesized by previously published procedure from PG by subsequent steps of mesylation, azidation and Staudinger reduction. The reductive amination of the polyglycerolamine with a large excess of maltose or *N*-acetylglucosamine was carried out with a borane-pyridine complex as a strong reducing agent in a sodium borate buffer, yielding *N*-acetyl-glucosamine linked polyglycerolamine (PG_10_-GLNC, **14**) and maltose-modified polyglycerolamine (PG_10_-Mlt, **15**). The ^1^H-NMR spectrum of **14** showed signals in two characteristic regions: from 2.5 to 4.4 ppm (PG protons overlapped with those from sugar) and from 5.0 to 5.3 ppm (anomeric proton from maltose). On the other hand, ^1^H-NMR spectrum of **15** displayed a broad signal in the range of 1.9–2.2 ppm (CH_3_ group of the *N*-acetylgluocosamine) followed by highly overlapping signals broadened from 2.5 to 4.5 ppm (proton from PG backbone overlapping with those from sugar units). The reductive amination reaction of the reducing unit of the parent (oligo-) saccharide yields a noncyclic unit partly restricted in mobility by its neighborhood to the PG scaffold and, therefore, generating signals with different extents of broadening. Additional broadening and splitting should be caused by different stereochemistry and by the substitution pattern of the neighboring amine nitrogen. Nevertheless, for compound **15**, signal at 22 ppm and for **14**, at 100 ppm in ^13^C spectra after dialysis indicated the immobilization of the saccharide residue onto the PG scaffold. It was difficult to conclude whether one or two units of sugar residues have been added per amino group, however from ^1^H-NMR, the degree of functionalization was calculated which was typically within the range of 48% for **14** and 54% for **15**.

### 2.5. Attaching N-α-BOC-Histidine to Polyglycerolamine: BBB Targeted Nanocarriers

**Scheme 4:**
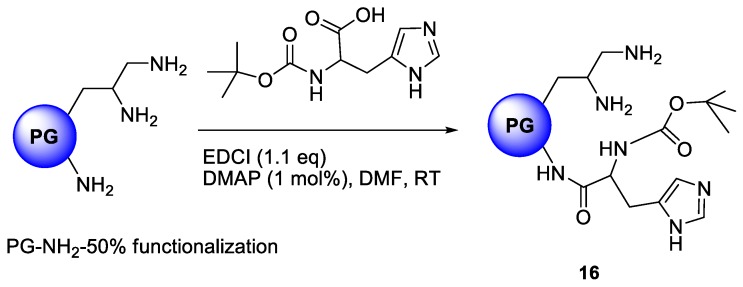
Coupling of *N*-α-BOC-histidine to polyglycerolamine.

Brain microvascular endothelial cells (BMEC) are the major structural and functional components of the BBB that maintain the homeostasis of the central nervous system. The plasma membrane of BMEC has been shown to be the site of several carrier-mediated transport systems including those for glucose, monocarboxylic acids and amino acids [45,46]. Xiang et al., provided evidence for there being at least two pathways for l-histidine uptake into isolated choroids plexus, an Na^+^-independent and an Na^+^-dependent process. Hikichi et al., studied the stereoselective BBB transport of histidine using rat brain BMEC and reported the process to be saturable [47]. Their results suggested that l-histidine is actively taken up by a carrier-mediated mechanism into the BMEC with energy supplied by Na^+^. Considering these earlier data, we have immobilized a histidine analogue, *N*-α-BOC-Histidine was immobilized onto polyglycerolamine (50% amino functionalized) to yield compound **16** (PG_10_-His) in an attempt to utilize the Cu complexing capability of both polyglycerolamine and imidazole ring system of the histidine moiety while exploiting the affinity of histidine nucleus towards System-L and System-N transporters in the BBB. In this system, *N*-α-BOC-Histidine serves as the targeting modality to target histidine transport receptors. The reaction chemistry is presented in Scheme 4.

*N*-α-BOC-histidine was coupled to polyglycerolamine by using EDCI as the coupling agent in the presence of catalytic amount of DMAP in DMF-water (1:1) mixture. An overnight dialysis in methanol yielded pure compounds in 78% yield. ^1^H-NMR conclusively proves the immobilization of the derivatives onto PG scaffold where the nine protons from BOC signals resonate at 1.09 ppm while two protons from the imidazole rings of histidine nucleus appear at 6.52 and 7.84 ppm. The degree of functionalization was adjusted to 20%, which resulted in water-soluble product.

### 2.6. Synthesis of FITC-Labelled PG_10_-TMEDA System

Compound **3f** (approximately 70% level of functionalization) was tagged with FITC in the presence of dibutyl tindilaurate as catalyst. Extensive dialysis in MeOH yielded the desired FITC labeled **3f** (FITC-PG_10_-TMEDA) for cellular uptake studies. UV-Vis spectrophotometric methods and ^1^H-NMR were used to characterize the labeled product.

### 2.7. Cu-Encapsulation by Synthesized hPG-Derived Nanocarriers: UV-Visible Spectroscopic Investigation

**Figure 2:**
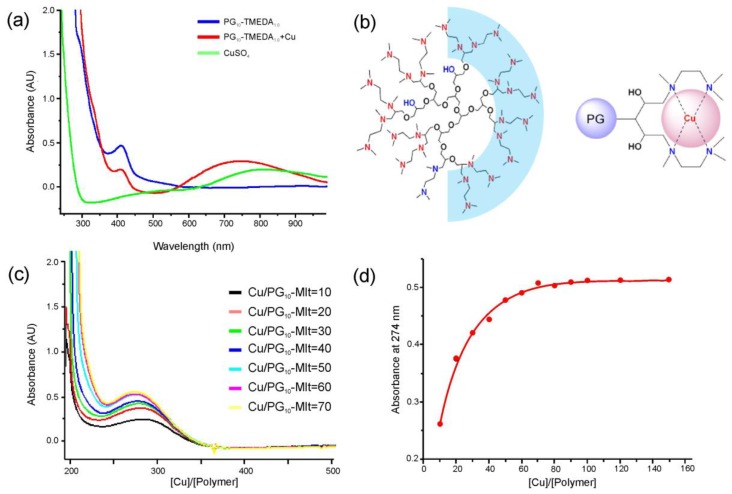
(**a**) Complexation of Cu^2+^ion by PG_10_-TMEDA_1.0_ system **3f** as indicated by shift of absorbance band at shorter wavelengths compared to that for free CuSO_4_ (**b**) **Idealized structure** of compound **3f**, and the possible structurea of the complex (**c**) Complexation of Cu^2+^ ion by PG_10_-Mlt **14** can be followed by gradual increase of absorbance at 273 nm. Cu/Polymer ratio of 10–70 is presented (**d**) Two distinct region is present before and after maximum Cu loading by the PG_10_-Mlt **14** system which diverges at [Cu]/[Polymer] = 50.

The primary objective of designing these synthetic constructs was to use them for complexing and delivering Cu ion across cell membranes. To this end, the hPG-based architectures were first tested for their Cu-complexing ability. UV/Vis spectroscopy is a method that is extensively used to investigate qualitative as well as quantitative changes in the coordination sphere of transition-metal complexes [48,49,50,51]. In the case of dendrimers, it was shown that dendritic structures can only encapsulate a maximum number of metal ions per molecule [52]. The maximum metal loading of the dendrimer and the value of the [metal ions]/[dendrimer] ratio determines the size of the formed nanoparticles [53]. In our case, a similar approach to investigate the metal loading capacity by PG based Cu encapsulating nanostructures was undertaken. Cu^2+^ encapsulated systems were prepared by mixing equal volume of aqueous CuSO_4_ solution with respective polymer solution. An incubation time for metal uptake was standardized to be 24 h. The encapsulated Cu^2+^ ion was not reduced after complexation to form true nanoparticles since the therapeutic efficacy needs the Cu^2+^ ion to be delivered in ionic form. The evidence for Cu^2+^ encapsulation by the respective PG based Cu^2+^ encapsulating constructs resides in the fact that, in pure aqueous solution copper sulfate forms a light blue (aqua) copper complex, [Cu (H_2_O)_6_]^2+^, which has a wide absorbance maximum at 810 nm region. On the formation of a complex between the amine moieties of polymer scaffold, this absorbance maximum will be shifted to shorter wavelength. At this wavelength, a continuous variation plot of Cu to polymer ratio will provide the maximum number of Cu ions that can be encapsulated per mole of nanocarrier [54,55]. As a general procedure, to assess maximum metal-cargo capacity of dendrimers and hyperbranched polymers, an aqueous solution of the individual nanotransporter (at various concentrations calculated according to the M_n_ value) was mixed with an aqueous solution of Cu^2+^ to obtain a distinct molar [Cu^2+^ ions/nanotransporter] ratio within the range of 0–100. As a representative example, Figure 2a illustrates the complexation of copper ions with PG_10_-TMEDA_1.0_ system. A more detailed biophysical and biochemical evaluation of Cu-complexing capacity of this candidate and related molecular species has been communicated in our earlier publications [12,13,14]. In the absence of a polymer with complexation capabilities, Cu^2+^ exists primarily as [Cu (H_2_O) _6_]^2+^ in aqueous solutions, which gave rise to a broad, weak absorption band at 810 nm associated with a d-d transition (ε ~ 10). In the presence of **3f**, λ_max_ for the Cu^2+^ d-d transition was shifted to 735 nm. In addition, a strong ligand-to-metal-charge-transfer (LMCT) transition appeared at 280–300 nm regions (Figure 2a). With higher ratio of Cu^2+^ to polymer, the spectrum shows a tendency to shift towards the longer wavelength. This change in UV-Vis spectrum allows following the Cu^2+^ ion binding with different hyperbranched polymer systems bearing nitrogen atoms. In this experiment, the absorbance at λ_max_ at 735 nm increased with increasing [Cu^2+^]/[nanotransporter] ratio until a critical value is reached, above which the absorbance increased only slowly. The shift in the absorption maximum at 735 nm is mainly due to the complexed Cu ion co-ordinated within the four nitrogen system of TMEDA structure across the polymeric scaffold. Figure 2b represents the general architecture of such TMEDA complexes with Cu^2+^, where the metal ion is bound between the four nitrogen atoms of TMEDA moiety of compound **3f**. A different UV-Vis absorbance pattern was observed when compound PG_10_-Mlt **14** or PG_10_-GLNC **15** was titrated with Cu^2+^. Figure 2c,d shows the absorption spectra of **14** in the presence of increasing amount of Cu^2+^. The nanocarriers themselves do not absorb light significantly above 250 nm. In the presence of Cu^2+^, a new peak at appears at 273 nm (Figure 2c for PG_10_-Mlt system). This can be attributed to the ligand-to-metal charge-transfer (LMCT) transition of the Cu^2+^ encapsulated complexes [54,55]. This LMCT band were obtained when increasing amount of Cu^2+^ was added to PG_10_-Mlt system in water.

Comparative studies have been undertaken which revealed no interaction between maltose with Cu^2+^. Therefore it can be assumed that the amino groups within the polymeric scaffold are responsible for complexing Cu^2+^. Because of the low absorbance of d-d transitions (less than 0.12) at about 700 nm, which is also associated with high signal-to-noise ratio, the LMCT transition at 290 nm was used for quantitative evaluation of Cu^2+^/polymer complex. Figure 2d shows the absorbances of PG_10_-Maltose system as a function of Cu^2+^ concentration. At low Cu^2+^ concentration, a linear increase in absorbance at 274 nm was observed. In this region, an effective complexation takes place between Cu^2+^ ions and hPGs. This gradual increase of absorbance eventually diminishes into an approximately linear region. Here practically no complexation between Cu^2+^ and polymers takes place. The intersection point of these two regions can be estimated as the maximum molar capacity of these carbohydrate modified polyglycerolamine systems to load Cu^2+^ ions. 

For PG_10_-His system **16**, a shoulder band at 290 nm, which gradually increases with increasing Cu^2+^ concentration, was assigned as LMCT transition signals and similar procedures as mentioned above was followed to determine the Cu^2+^-loading capacity of these nanocarriers. The high signal-to-noise ratio of Cu^2+^/carrier complexes from 650 to 700 nm, associated with the problem of overlapping of the LMCT region by the structural features of the carriers themselves restricted the UV-Vis spectroscopic application to determine maximum metal-encapsulating capacity of PG_10_-NF135 **11** and PG_10_-KR 455 **12** systems. For these carriers, isothermal titration calorimetry (ITC) was used as a method of choice to investigate their encapsulation/complexation behaviour. 

### 2.8. Thermodynamics of Cu-Ion Encapsulation by hPG Nanoconstructs: Isothermal Titration Calorimetry (ITC)

Isothermal titration calorimetry (ITC) has proven its efficiency for studying thermodynamic and kinetic properties of macromolecular interactions due to its ease of application and high level of accuracy. ITC technique was used to follow the enthalpic interactions between hPG-derived nanotransporter and Cu^2+^ ions, which in turn reflect the strength and extent of metal encapsulation properties of the selected species. The energetics of encapsulation is critically important for designing Cu-ion delivery systems as it governs the stability of the complex in fluctuating in vivo environment before it reaches the target tissue. Encapsulation of all nanotransporters was analysed by measuring the heat change during the titration of Cu^2+^ to nanotransporter solutions. The heat released or absorbed during the titration process is in direct proportion to the amount of binding between the macromolecule and the metal ion. In this experiment, isothermal titration calorimetry was performed in both orders, i.e., nanotransporters added to Cu^2+^ ion solution and vice-versa to investigate the encapsulation event quantitatively. It was found that, adding Cu^2+^ ion solution from the syringe to the nanotransporter solution in the cell gives a better representation of the saturation process of the polymers by Cu^2+^ ions. A series of concentration experiments has been done for each of the nanotransporters to fix the experiment parameters including the concentration of the hPG derivatives and the metal ion. 

**Figure 3:**
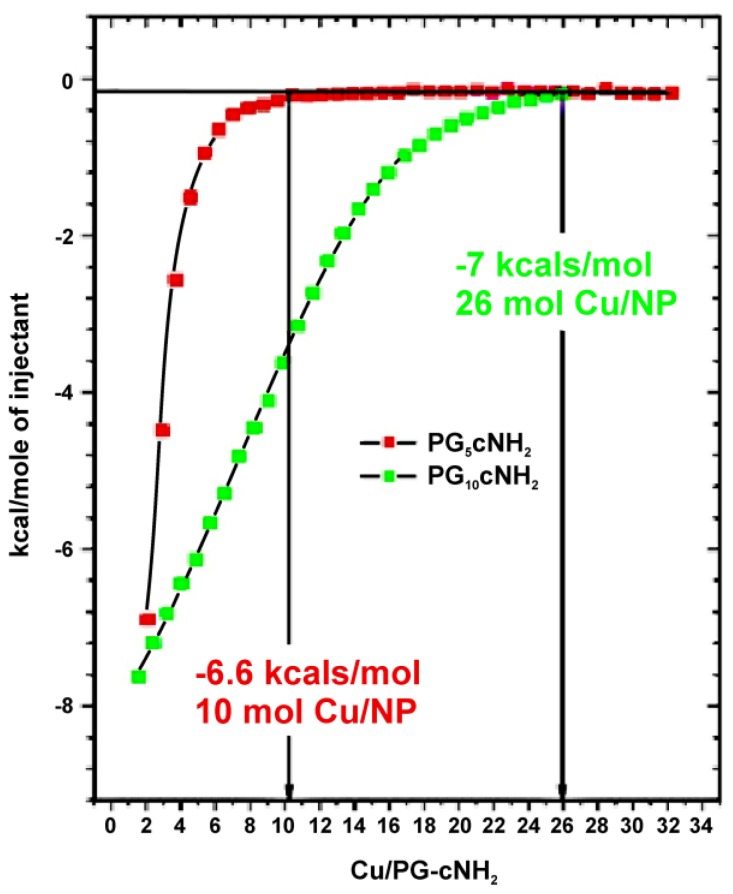
Binding isotherm of Cu^2+^ interaction with PG_5_-cNH_2_ **8a** and PG_10_-cNH_2_ **8b** systems. X-axis shows the amount of heat (kcal mol^−1^) released upon encapsulation of Cu by the nanocarriers.

Figure 3 illustrates the isothermal titration calorimetric trace for PG-cNH_2_ architecture **8**. Encapsulation stoichiometry of Cu^2+^ ions by these nanocarriers was found to be a function of the molecular weight of the PG core. Resulting binding isotherm representing the interaction of Cu^2+^ ions to this nanotransporter was hyperbolic in nature with an initial rapid release of heat in the negative side of titration baseline indicating exothermic interaction between Cu^2+^ ions and the nanocarriers. 

The enthalpic values, as illustrated in the figure, finally reached a plateau, which indicated the end-point of titration confirming that all or at least most of the accessible binding sites of the macromolecules have been saturated with the ligand. The negative value of apparent change in enthalpy (*ΔH_app_*) indicated the predominant effect of charge-interaction in binding event. Different statistical and qualitative approaches were undertaken to analyse the binding isotherm resulting from calorimetric titration. In principle, the difference between the amount of energy liberated/absorbed at the onset of titration and that at the end of the saturation process, generally gives the first approximation of binding energy of interaction. Stoichiometry of binding can be calculated from the inflection point of the titration curve. The stoichiometry can also be evaluated from the intersection points of different kinetic regions within the binding isotherm before the saturation/plateau level of interaction is reached. From exothermic heat release profile, it can be concluded that both core-functionalized polyglycerolamines PG-cNH_2_ **8** was complexing Cu^2+^ ions. PG_10_-cNH_2_ architecture was able to complex 26 mole of Cu ion per mol of polymer, whereas PG_5_-cNH_2_ was encapsulating only 10 mole of metal ion per mole of the nanocarrier. The heat of complex stabilization was almost of similar order for both variants, which is within the range of −6.5 to −7 kcal mol^−1^ indicating considerably stable complexes between Cu^2+^ ions and the polymers. The binding isotherms of these two polymeric systems however showed that PG_5_-cNH_2_ is saturated with Cu^2+^ ions at a faster rate than that of its high molecular weight counterpart. The observation might be due to easier accessibility of the metal ions towards the linear amino groups for low molecular weight core. The change of complexation enthalpy as a function of [Cu^2+^] to [nanocarrier] ratio followed a similar pattern for other nanocarrier systems. The exothermic isotherms can be mainly attributed to the charge transfer based complexation between the amine groups of the molecule and Cu^2+^ ions. Of the two carbohydrate-coupled polyglycerolamines, PG_10_-Mlt **14** showed different energetic behavior on its interaction with Cu^2+^. At the onset of titration, endothermic signals were generated which were gradually converted to exothermic heat flow (data not shown). The observed facts could be due to two separate energetic processes occurring during complexation. Similar effect was also reported previously where introducing larger oligosaccharide units onto the host polymer changes the binding mechanism of guest molecule [56]. 

**Figure 4:**
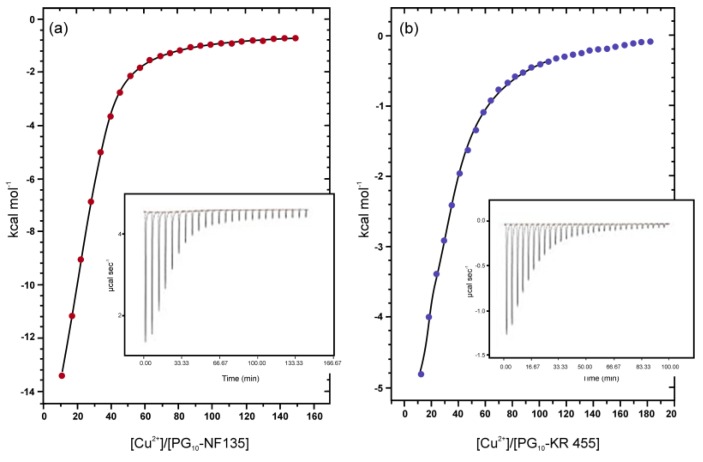
Binding isotherm for Cu^2+^ interaction with (**a**) PG_10_-NF 135, **11** and (**b**) PG_10_-KR 455, **12**.

PG_10_-NF 135, **11** and PG_10_-KR 455, **12** systems were analyzed micro-calorimetrically in the same manner to evaluate their Cu-complexing ability. Figure 4 shows the binding isotherms of these systems with Cu^2+^ ions. As observed from Figure 4a, approximately 13 kcal/mol of energy has been liberated on interaction of Cu^2+^ with PG_10_-NF 135 system, which indicates the formation of considerably strong complex. On the other hand, Cu^2+^-PG_10_-KR 455 complex stabilization energy was only within the range of 5 kcal/mol (Figure 4b). Intersection of the linear regions of binding isotherms affords the maximum Cu^2+^-loading efficiency of the nanocarriers. The figure also shows that PG_10_-NF 135 (**11**) can encapsulate ~45 mol of Cu/mol of carrier, while PG_10_-KR 455 (**12**) was able to bind ~55 mol of Cu/mol of carrier. 

Depending on the variation of molecular architecture, Cu^2+^ saturation level and complex stabilization energy varied with individual Cu^2+^ encapsulating systems. The binding isotherms of the different nanocarriers with Cu^2+^ ion allowed the determination of maximum [Cu^2+^]/[nanocarrier] ratio using the inflection method as per earlier published procedure [56]. Saturation stoichiometry of the nanotransporters towards Cu^2+^ generated by analysing the titration curves is presented in Table 3. The Cu-complexing capacity of compound **3f** (i.e., PG_10_-TMEDA_1.0_, fully functionalized) has been published in an earlier report [13]. Here we show that, compound **3f**, functionalized with TMEDA moiety at 50% and 10% functionalization level, also showed similar trend of Cu-encapsulation characteristics as per ITC and UV-Vis measurements.

### 2.9. Surface Charge Characteristics of Cu-Loaded hPG Nanocarriers

**Figure 5:**
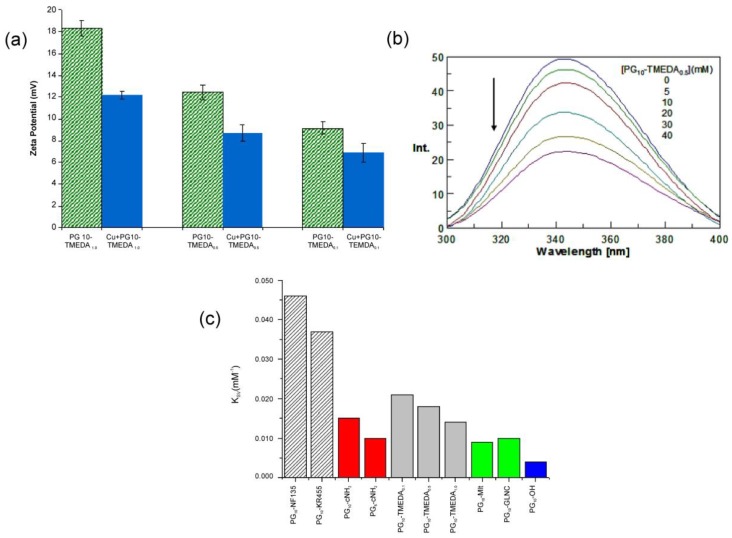
(**a**) Comparative zeta potential of PG_10_-TMEDA system alone and in the absence and presence of Cu^2+^. Gradual decrease of zeta potential is observed with decreasing functionalization level of PG core by TMEDA moiety (**b**) Fluorescence spectra of BSA (emission maximum at 341 nm) in the presence of increasing concentration of PG_10_-TMEDA_0.5_ (**c**) Stern-Volmer constant (K_SV_) of the nanocarriers with respect to PG (PG_10_-OH).

The surface charge, or zeta potential of nanocarriers governs a wide array of their biological activity, including cellular permeability, biocompatibility, systemic stability, enzyme interaction, metabolic degradation and toxicity. Hence, we have investigated the surface charge of the synthesized Cu-nanocarriers either alone or in Cu-encapsulated form. Zeta potentials of the PG_10_-TMEDA nanocarrier solution in PBS of pH 7.4 were measured for different degree of functionalization of the construct. To assess the effect of Cu^2+^ on surface charge of PG_10_-TMEDA systems, the nanocarriers were loaded with maximum stoichiometric amount of Cu (estimated from ITC and UV-Vis titration). For optimal loading of Cu, after incubation, free metal ion was removed from the polymer-encapsulated species by dialysis until a stabilized value of absorbance at 735 nm is obtained for the retentate solution. The freeze-dried, PBS resuspended solution of nanocarriers was subjected to zeta potential measurement. In case of PG_10_-TMEDA systems protonation may occur at all nitrogen atoms and like amine terminated PAMAM dendrimers, the surface of the polyglycerolamines will be positively charged and zeta potentials will gradually increase with increased percentage of cationic groups. Typical results from the zeta potential measurements are illustrated in Figure 5a for PG_10_-TMEDA system **3f** in buffered aqueous solution. It is clearly observed that at pH 7.4, these systems were positively charged and surface charge increases with increasing degree of functionalization of PG scaffold with TMEDA moiety from +10.4 mV to as high as +18.2 mV. Loading PG_10_-TMEDA systems with saturating concentration of Cu load (as measured from ITC/UV calculations) decreases the zeta potential of the Cu-encapsulated complex. For example, PG_10_-TMEDA_1.0_ **3f** system exhibits surface charge of +18.2 mV, which is decreased to 12.1 mV upon encapsulation of Cu^2+^ (Figure 5a). This charge neutralization of the carrier system by Cu^2+^ proves the location of the metal ion adjacent to TMEDA shell systems of the carrier molecule with probable involvement of the charge transfer mechanism. The higher the degree of functionalization, the stronger is the charge neutralization, most likely due to higher stoichiometric load of Cu ion within the polymer scaffold. Also considering the fact that, CuSO_4_ does not have any surface charge, the colloidal electrostatic charges of the resulting solution over entire [Cu^2+^]/[PG_10_-TMEDA] functionalization range is essentially being contributed from the Cu-encapsulated PG_10_-TMEDA system. Surface charge properties of PG-cNH_2_ **8** system were found to be a function of molecular weight of the PG core. PG_10_-cNH_2_ derived from 10 kDa PG scaffold exhibit higher zeta potential (16.4 mV) than PG_5_-cNH_2_ species with 5 kDa PG core (10.4 mV). For carbohydrate-modified carriers, surface charge was substantially low. 

Table 4 summarizes the zeta potential values of the studied nanocarriers:

### 2.10. Interaction of Cu-Ion Encapsulating Systems with Plasma Albumin

The effectiveness of drug or delivery system candidates depends heavily on their interaction with naturally occurring plasma proteins within the systemic circulation. This is particularly critical when the polymeric architectures possess high surface charge, molecular weight, and have tendency to interact with biomacromolecules in blood due to their surface properties. In general, the extent of interactions between bioactive molecules and serum is quantified by Stern-Volmer constants, reflecting tryptophan fluorescence quenching efficiency of the ligands, where the amino acid being the integral part of BSA construct. Furthermore, these interactions are measured in terms of binding constants or binding associations. Reported studies have been directed to realize the interactions of polyamidoamine (PAMAM) dendrimers (fatty acid free and loaded with fatty acids) with serum albumins (SA), for their physiological significance in developing nano-carrier systemic delivery system [57]. In our case, fluorescence spectroscopic studies were carried out in phosphate buffered saline solution. Emission spectra were recorded from 300 to 440 nm after excitation at 295 nm. In buffered aqueous solution, BSA exhibits a characteristic emission spectrum of the tryptophan fluorophore, with a distinct peak maximum at around 340 nm. In order to investigate the interaction between dendritic polyglycerols and BSA, changes in the intensity of the emission spectra of BSA upon addition of hPG-constructs were monitored. As can be seen from the spectra in Figure 5b, addition of PG_10_-TMEDA_0.5_ **3f**–**b** to BSA in buffer led to a significant decrease in the intensity. When the PG_10_-TMEDA_0.1_ **3f**–**c** carrier was added to the solution of BSA, the spectra showed only a slight decrease in the fluorescence intensity; therefore suggesting much weaker binding interactions to BSA indicating the interaction is operating mainly through the TMEDA groups coupled to PG. On the contrary, in the experiment with PG_10_-Mlt, emission spectra of BSA were less affected by the presence of the attached maltose units onto PG scaffold (data not shown). The distinct decrease in the fluorescence intensity, as in Figure 5b, upon addition of polymers can be utilized in studying the interaction of PG based carriers with BSA quantitatively by using classical Stern–Volmer equation [58,59,60]:

(1) $$F_{0}/F=1+k_{q}\times \tau_{0}\times \left[Q\right]=\;1+K_{SV}\times \left[Q\right]$$

where *F*_0_ and *F* are, respectively, BSA fluorescence intensities in the absence and presence of polymer, *K*_SV_ is the Stern–Volmer quenching constant, [Q] is the concentration of polymer, *k*_q_ is the bimolecular quenching constant, and τ_0_ is the lifetime of the fluorophore in the absence of quencher. If diffusion controlled, collisional quenching process is assumed, Equation (1) can be applied to properly fit the experimental data. 

The slope of Stern-Volmer equation equals to *K*_SV_ and usually reveals the accessibility of the polymers to the albumin fluorophore, hence the interaction and potential protein binding capacity of the quencher [59]. More precisely, a linear plot is generally indicative of a single class of fluorophores, all equally accessible to the quencher molecules. Typical results of Stern-Volmer constants of other carriers are presented in Figure 5c. Spectroscopic results imply that the electrostatic interaction is the main driving force for binding of these nanocarriers to negatively charged BSA. For example, if the PG_10_-TMEDA **3f** systems (light blue colour bars) are considered, it is clearly apparent that the extent of interaction which is quantified by Stern-Volmer constant, decreases with decreasing functionalization level of PG hydroxyl groups by TMEDA moiety. Similar trend can be observed with PG-cNH_2_ system, where the *K*_SV_ value clearly depends on molecular weight of the PG core. Attachment of sugar residue onto PG scaffold essentially reduces the interaction with BSA. On the contrary, PG_10_-NF 135 **11** and PG_10_-KR 455 **12** system exhibited substantially high interaction with BSA probably due to the presence of heterocyclic ring, which facilitates non-specific attachment with BSA in addition to hydrophobic mediated interactions.

### 2.11. Cellular Toxicity and Uptake of Representative hPG-Derived Nanocarriers

**Figure 6:**
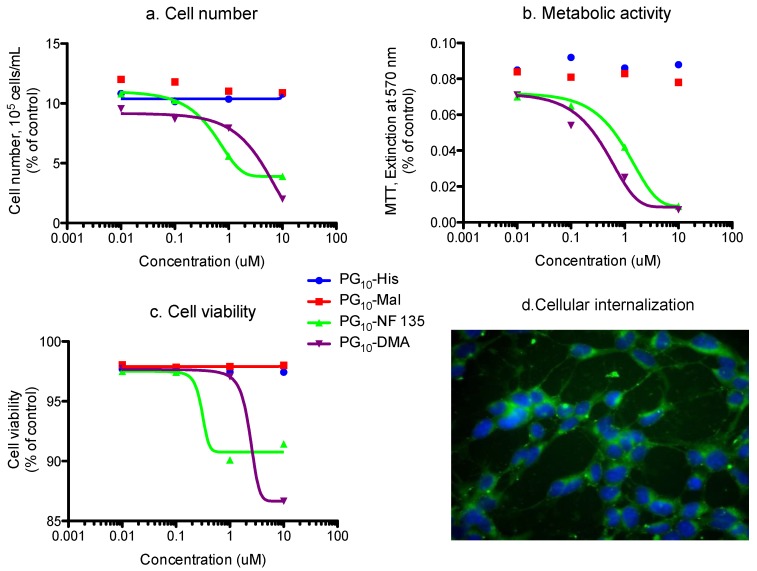
Cellular studies with U-937 cell-line treated with different hPG derivative Clock-wise: (**a**) Cell number (**b**) metabolic activity; and (**c**) Cell viability (**d**) Presence of FITC-labelled compound **3f** in cytosol after 30 min incubation with SY5Y cell lines.

Cellular toxicity studies of the architectures in human hematopoietic cell line U-937 are shown in Figure 6. A set of four parameters were considered for the evaluation of the Cu^2+^ nanocarrier systems: (a) Cell number; (b) MTT-test for metabolic activity (c) Cell viability and (d) Apoptosis in terms of cell diameter with respect to dexamethasone control. In all the cases, PG_10_-His **16**, PG_10_-Mlt **14** and PG_10_-GLNC **15** showed substantial cell viability, unaltered cell diameter, normal metabolic activity and significant cellular biocompatibility than PG_10_-DMA **3a**, PG_10_-NF135 **11** and KR455 **12**, and architectures. Figure 6 illustrates the representative cell-toxicity study obtained with PG_10_-GLNC system **15**. In addition to control, we have added dexamethasone to evaluate the effect of a known cytotoxic agent within the same concentration as that for our test samples. As evident from the figure, the number of viable cells, metabolic status and cell viability in general were virtually unaffected by PG_10_-GLNC (**15**) or by PG_10_-His systems (compound **16**). This might be due tohe biocompatibility of *N*-acetylglucosamine or histidine analogue that has been attached onto polyglycerolamine scaffold. On the other hand, PG_10_-KR 455 **12** system was found to be highly toxic to the U-937 cell line as illustrated in supporting information, and so was dimethylamine-substituted hPG (compound **3a**). Cytotoxicity data of compound **3f** has been published in our earlier report [13]. This observation might be attributed to extremely strong Cu-binding efficiency of these systems, which possibly depleted essential metal ions from cellular microenvironment. We have also studied a short-term incubation of PG_10_-TMEDA **3f** system with 90% level of functionalization and PG_10_-cNH_2_ with living neuronal SY5Y cell lines. When the cell lines were subjected to Cu loaded and FITC-labelled PG_10_-TMEDA system, green fluorescence of FITC was observed around the DAPI stained nucleus on superimposing image, confirming the cellular uptake of the carrier system within the cytoplasm (Figure 6d) starts taking place within 30 min as observed in confocal microscopy based experiments. We have also reported a more extensive cellular and biochemical property of this system, which showed that, not only PG_10_-TMEDA systems were capable of internalization inside cytosol, they were also able to engage with cytoplasmic metal processing machinery [13,14]. In a similar way, core-functionalized polyglycerolamine, PG_10_-cNH_2_ **8a** tagged with FITC was investigated for cellular uptake in SH-SY5Y cell-line, and the dye-labelled nanoparticles were found to be associated to the nuclear membrane after 30 min of incubation period (data not shown). Since these cationic constructs do not contain any receptor-specific ligand, the observed cellular uptake of is most likely triggered by non-specific endocytosis, followed by endosomal escape through proton-sponge effect. 

## 3. Discussion

We found that, bare hPGs do not encapsulate Cu ion to a significant extent. Modification of PG core is required to introduce Cu-encapsulating modalities within the molecule. This has been achieved either by activating the PG hydroxyl group to an efficient leaving group, following nucleophilic substitution with different dialkylamine moieties or, by step-wise conversion of PG hydroxyl groups to primary amines. Cu-encapsulating moiety can be strategically localized either within the core, or introduced statistically throughout the molecule. The complex formation of hPG-derivatives with the metal is believed to take place through the displacement of water molecules surrounding Cu^2+^ ion in aqueous solution. Along with typical mechanism of charge transfer from N-atom of the polymer towards Cu^2+^ ion, it is also assumable that there is an enthalpic-entropic compensation in the formation of such complexes. The role of entropic part in addition to enthalpic component in host-guest interaction [61,62,63] is a well-documented phenomena particularly in case of charge-interactions [64]. Encapsulation of a guest species, Cu in this case, resulted in reduced rotation of bonds and higher ordered structures applicable to all tested candidates. The partial/total loss of hydrate shell around Cu ion set water molecules free which increased entropy and might compensate for the loss of rotational freedom after encapsulation [65]. We have reported earlier, that compound **3f**, at full functionalization level, not only showed high affinity encapsulation of Cu^2+^, but also a pH-dependent release in aqueous environment [13]. The stability of all tested complexes at pH 7.4 is important to retain the integrity of the complex in the blood stream before accumulation into target tissues. On the other hand, gradual release of Cu in acidic environment will be of critical importance in the liberation of Cu ion within the cytosol after endocytosis. 

hPG-derivatives with primary amine groups at the core (compounds **8a** and **8b**), showed Cu-encapsulation efficiency as a function of the MW of the core. Heat of complex stabilization was almost similar for both molecular weight variants, however, the rate of saturation of nanocarriers were much faster in case of high MW PG-species **8** than their low MW counterparts (Figure 3). This can be attributed to the higher accessibility Cu towards the linear amino groups in case of low MW polyglycerolamine. Similarly, hPG-derived compounds, particularly those conjugated with bioactive ligands such as maltose, *N*-acetylglucosamine and histidine showed increased toxicity tolerance for free Cu (Figure 6) in the cell. Attaching carbohydrate functionalities to the core, i.e., maltose and *N*-acetylglucosamine, as in compounds **14** and **15** respectively, is a well-documented approach to increase compartmentalization of guest molecules and to exert biocompatibility. In this project, addition of carbohydrate fragments was envisioned to stabilize the Cu^2+^ complex formed within the polyglycerolamine scaffold. These architectures were found to be the most non-toxic of all the synthesized architectures in U-937 cell-line in terms of cell-viability, metabolism and apoptosis. Improvement of biocompatibility scenario through incorporation of carbohydrate shell has been reported before [56]. Attachment of bispicolylamide derivatives to PG improved the Cu^2+^ binding ability of PG based nanocarriers to a considerable extent (Figure 4). PG_10_-NF135 **11** was found to be the strongest Cu^2+^ complexing nano-architectures among the synthesized candidates in terms of binding energy. Compound **11** was found to accommodate ~55 moles of Cu/mol of polymer with an energy range of 13 kcal mol^−1^. Unfortunately, the bispicolylamide derivative immobilized PG nanocarriers exhibited substantial level of toxicity compared to control. This could be attributed to strong chelating ability of bispicolylamide towards Cu, and also to other metals that might have damaged normal metal homeostasis within cellular environment. Similar results were also reflected in binding experiments of nanocarriers with serum albumin. Stern-Volmer constant (*K*_SV_), which is considered as a quantitative extent of interaction with BSA with a given quencher molecule, decreased gradually in the following order: PG_10_-DMA, **3a** > PG_10_-NF135, **11**, PG_10_-KR 455, **12** > PG_10_-TMEDA_1.0_, **3f** > PG_10_-His, **16**, PG_10_-TMEDA_0.5_ **3f**-**b** > PG_10_-Mlt, **14**, and PG_10_-GLNC, **15**. When neuronal cells, such as SY5Y, were treated with FITC-tagged **3f** derivatives, FITC fluorescence from the nanocarriers were visualized near the DAPI stained nucleus indicating that the systems were uptaken by the cells through non-specific endocytosis followed by endosomal escape. In case of core-functionalized polyglycerolamine, accumulation of the nanocarriers on or near nuclear membrane was evident. 

The experimental significance of this work principally indicates that, it is possible to construct multivalent hPG-based ligand for spontaneous encapsulation of Cu ion to generate biocompatible Cu-complexing macromolecular constructs. The strength and complexation property can be optimized by selective functionalization of the hydroxyl groups of hPG scaffold by different structural modalities, which in turn also governs cellular uptake and toxicity of the nanocarriers. The optimized hPG-derived architectures can find potential application in addressing disease and nutritional deficiency conditions where exogenous administration of Cu is required.

## 4. Experimental Section

### 4.1. Materials

PG (*M*_n_ = 10,000 g/mol) synthesized from TMP starter, MWD = 1.7) was prepared by the Haag group according to a previously published procedure. All PG samples were concentrated and dried under vacuum (50 °C, 1 × 10^−2^ mbar) until loss of weight was lower than 0.025 g per 1.0 g of the dried sample in 5 h drying periods. This process was accepted as a standard procedure followed also in previous works. Commercially available chemicals from standardized sources have been used and used as delivered. Solvents were purchased as reagent grade and distilled if necessary. Anhydrous solvents were either purchased as ultra dry solvent from Acros Organics^®^ (Fisher Scientific, Im Heiligen Feld, Schwerte, Germany) or received from solvent purification system. The following spectrometers were used for recording ^1^H-NMR and ^13^C-NMR spectra: DRX 500 and AMX 500 spectrometers, (Bruker, Billerica, MA, USA). Typically, 10–30 mg of compound was used for recording ^1^H-NMR while 50–100 mg of compounds were required for ^13^C-NMR. Deuterated solvents were used as standardized procedure. All spectra were recorded at r.t. and were analyzed with Win NMR^®^ software (Bruker, Billerica, MA, USA). Benzoylated cellulose membrane purchased from Sigma-Aldrich (Munich, Germany), MWCO = 1000 was used to perform dialysis. Typically, dialysis was carried out for 24 h with 1 L of solvent that was exchanged after first 6 h of the process. Size exclusion chromatography (SEC) was performed with Sephadex LH 20 or Sephadex G 25 from GE Healthcare (Little Chalfont, UK). The material was activated by swelling in the respective eluent prior to performing chromatography. In case of using CHCl_3_, the beads were activated in the presence of 10% ethanol. TLC was performed on aluminium sheets with silica (corn size 60) and fluorescence marker (F_254_) (Merck, Darmstadt, Germany). Flash column chromatography was performed on Merck silica (corn size 60).

### 4.2. UV-Vis Spectroscopy

UV/Vis spectra were recorded with a S-3150 instrument (Scinco, Seuol, Korea) range: 190–1100 nm, resolution 1024 points) in fast mode. Calibration was performed at 360.85 and 453.55 nm with holmium oxide glass. The spectra were recorded at r.t. and were evaluated with Labpro^®^ Plus from Scinco Co., Ltd., Microsoft^®^ Excel 2000 (Microsoft Inc., USA), and Origin^®^ 7.0 from Origin Lab Corporation (Redwood City, MA, USA).

### 4.3. Isothermal Titration Calorimetry (ITC)

A Microcal VP-ITC microcalorimeter (MicroCal, LLC, Northampton, MA, USA) was used to carry out the calorimetric experiments. Experimental parameters for titration experiments were: number of injections 34, cell temperature 30 °C, stirring speed 290 rpm, cell volume 1.43 mL, injection volume 8 µL, injection duration 16 s, spacing 300 s and filter speed 2 s and reference power 10 µcal s^−1^.

### 4.4. Zeta Potential Measurement

Zeta potential measurement were carried out on a Zetasizer Nano ZS analyzer with integrated 4 mW He-Ne laser, λ = 633 nm (Malvern Instruments Ltd., Malvern, UK). Doppler anemometry technique was used whereby electric field was applied across the sample solution. All measurements were carried out at 25 °C using folded capillary cells (DTS 1060). The medium was PBS (pH 7.4) and particle concentration used was 2 mg/mL.

### 4.5. Fluorescence Spectroscopic Studies

Fluorescence emission spectra were taken with a FP—6500 spectrofluorometer (Jasco, Deutschland, Pfungstadt, Germany) equipped with a thermostated cell holder, a DC-powered 150 W xenon lamp, a R 928 photomultiplier and a versatile slit system. For polymer-BSA interaction studies, emission spectra were recorded in from 300 nm to 440 nm after excitation at 295 nm. Both excitation and emission slits were set at 5 nm. The sample solutions used for fluorescence measurement were stirred thoroughly by using a laboratory vortex shaker and incubated for at least half an hour at 25 °C before measuring the fluorescence. All measurements were carried out at 25 °C.

### 4.6. Synthesis of Core-Functionalized Polyglycerolamine; Ketalization of PG: Protection of Terminal Diols ***4***


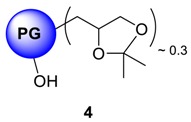


Ketalization of PG has been done following the already published procedure [22]. To a mixture of PG (10.0 g, 40.54 mmol of diol units, 1.0 eq.) and 0.4 mol (10.0 eq.) of acetone dimethyl acetal, 0.77 g (10 mol%) of PTSA was added. The reaction was allowed to run overnight under r.t. The crude product was diluted in chloroform and then extracted three times with saturated Na_2_CO_3_ solution to remove the remaining PTSA. The organic phase was dried over MgSO_4_. Dialysis in chloroform for 48 h was carried out to remove the traces of dimethylacetal and PTSA. The purified product was dried under vacuum to yield the polyketal as viscous, yellowish, transparent oil in 93% yield. Conversion: quant. (all hPG diols, 30% of total hydroxyl groups were converted). ^1^H-NMR (500 MHz, CDCl_3_): δ (ppm) = 1.17 (s, CCH_3_, ketal), 1.20 (s, CCH3, ketal), 3.28–4.06 (PG-backbone); ^13^C-NMR: (CDCl_3_): δ (ppm) = 26.1 (C-CH_3_, ketal), 27.6 (C-CH_3_, ketal), 64.2–80.1 (m, PG-backbone), 109 (C-CH_3_, ketal); IR (KBr) ῡ = 3440, 2980 (ketal; CH_3_), 2920, 2865, 1210 cm^−1^.

### 4.7. Mesylation of Ketal Protected PG ***5***


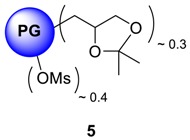


This reaction was carried out under an inert gas atmosphere and exclusion of water. Ketal protected PG (7.0 g, 32.7 mmol OH-groups, 1.0 eq.) in a three-necked 250 mL flask equipped with a drop funnel, thermometer and magnetic stirrer was dissolved in abs. pyridine (50 mL). The solution was cooled down to 0 °C by means of ice/NaCl bath and a solution of MsCl (3.0 mL, 4.5 g, 39.26 mmol, 1.2 eq.) in abs. pyridine (20 mL) was added drop-wise so that the temperature did not exceed 5 °C. The brown mixture was stirred for 16 h in the thawing ice bath. In case of full functionalization, after the reaction period, ice was added to the reaction mixture on which a dark brown solid precipitated, which was, after decantation of the liquid phase, washed with H_2_O, dissolved and dialyzed in chloroform to give a brown honey-like product in 87% yield. Conversion: quant. ^1^H-NMR (500 MHz, CDCl_3_): δ (ppm) = 1.19 (s, CCH_3_, ketal), 1.22 (s, CCH_3_, ketal), 3.13 (bs, -CH_3_), 3.28–4.26 (PG-backbone); ^13^C-NMR: (CDCl_3_): δ (ppm) = 27.1 (C-CH_3_, ketal), 29.6 (C-CH_3_, ketal), 35.3 (CH_3_), 64.2–82.1 (m, PG-backbone), 105 (C-CH_3_, ketal); IR (KBr) ῡ = 3040, 2980 (ketal; CH_3_), 2932, 2918, 2869, 2350, 1709, 1450, 1201) cm^−1^.

### 4.8. Procedure for the Synthesis of Ketal Protected Polyglycerolazide ***6***


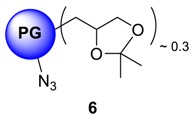


In a 250 mL one-necked flask with reflux condenser and magnetic stirrer was dissolved ketal protected *O*-mesylpolyglycerol (6.0 g, 18.06 mmol OMs-group, 1.0 eq.) in p.a. DMF. After complete dissolution of the mesylated derivative, NaN_3_ (5.87 g, 90.32 mmol, 5.0 eq.) was added to the flask and the resulting suspension was heated at 60 °C for 3 days behind a transparent security wall. After cooling, filtration delivered a bright yellow filtrate and a white residue of excess NaN_3_. The filtrate was concentrated in vacuo at temperature below 40 °C and only handled with plastic spatula to avoid explosive degradation of polyazide. In case of complete functionalization, the residue was dissolved in CHCl_3_ and extracted four times with water. The organic phase was dried over MgSO_4_ and concentrated in vacuo. Further dialysis in CHCl_3_ was carried out for 24 h to remove traces of DMF from the crude product. Extensive dialysis in CHCl_3_ for 24–48 h was carried out to yield polyglycerolazide as brown-paste like compound. Yield: 75%; Conversion: quant. ^1^H-NMR (500 MHz, CDCl_3_): δ (ppm) = 1.18 (s, CCH_3_, ketal), 1.20 (s, CCH_3_, ketal), 3.41–4.26 (PG-backbone); ^13^C-NMR: (CDCl_3_): δ (ppm) = 25.1 (C-CH_3_, ketal), 26.6 (C-CH_3_, ketal), 65.5–80.1 (m, PG-backbone), 107 (C-CH_3_, ketal); IR (KBr) ῡ = 2976 (ketal; CH_3_), 2870, 2350, 2101 (N_3_), 1465, 1234 cm^−1^.

### 4.9. Deprotection of Ketal Protected Polyglycerol Azide ***7***


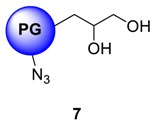


To a solution of ketal protected polyglycerolazide (1.77 g) in 25 mL MeOH, 2.0 g of Dowex-500 resin was added after activation. The mixture was stirred and heated at reflux for 18 h. The crude product was filtered, and the filtrate was concentrated, dried under vacuum and subjected to MeOH dialysis overnight. Yield: 70%; Conversion: quant. ^1^H-NMR (500 MHz, CDCl_3_): δ (ppm) = 1.81 (PG-starter), 3.41–4.26 (PG-backbone); ^13^C-NMR: (CDCl_3_): 50.1 (functionalized primary PG-groups) 65.5–80.1 (m, PG-backbone); IR (KBr) ῡ = 3300, 2802, 2365, 2101 (N_3_), 1234 cm^−1^.

### 4.10. Synthesis of Core-Functionalized Polyglycerolamine ***8***


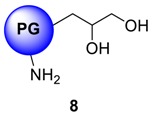


Deprotected polyglycerolazide (1.89 g, 7.79 mmol N_3_ group, 1.0 eq.) was dissolved in p.a THF (40 mL) in 250 mL one-necked flask. H_2_O (10 mL) and PPh_3_ (2.04 g, 7.79 mmol, 1.0 eq.) were added and N_2_ formation was observed. Volume of water was increased gradually by dropwise addition of water (30 mL) through a drop funnel to avoid precipitation of the partially reduced product. The reaction was continued until the azide signal at 2100 cm^−1^ is completely diminished. In most of the cases, depending on the level of functionalization of PG core, the complete reduction of N_3_ to NH_2_ group required 5 to 7 days. After that, the mixture was concentrated to in vacuo to smaller volume, CHCl_3_ was added and the phases were separated using a separation funnel. The aqueous layer was extracted with CHCl_3_ four times and then concentrated to dryness to deliver a brown honey-like product, which was dialyzed in MeOH. The purified product was not completely dried off MeOH, and stored under inert gas to avoid potential cross-linking. Yield: 65%; Conversion: quant.; ^1^H-NMR (500 MHz, CD_3_OD): δ (ppm) = 4.01–3.25 (PG), 2.86–2.73 (m, broad, NH_2_ functionalized-PG groups), 0.92 (PG-starter); ^13^C-NMR: (CD_3_OD): 58.3 (functionalized primary PG-groups) 67.5–76.1 (m, PG-backbone); IR (KBr) ῡ = 3441, 2872, 1455, 1208, 1104 cm^−1^.

### 4.11. Core Functionalization of PG Acetal with N^1^,N^1^,N^2^-Trimethylethane-1,2-Diamine ***9***


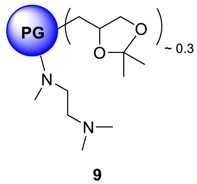


In a sealed tube, mesylated PG acetal (1.0 g, 2.10 mmol OMs group, 1.0 eq.) was dissolved in 10 mL of p.a. DMF. *N*^1^*,N*^1^*,N*^2^-trimethylethane-1,2-diamine (0.858 g, 8.40 mmol, 4.0 eq., 1.1 mL) was added slowly at RT and the resulting solution was heated at 120 °C for 4 days. At the end of the reaction, the tube was cooled to RT and DMF was removed by cryo-distillation. The residue was dissolved and dialyzed in MeOH for 48 h to give the dark brown honey-like product. Yield: 64%; Conversion: quant (all hPG diols, 30% of total hydroxyl groups were converted); ^1^H-NMR (500 MHz, CD_3_OD): δ (ppm) = 4.25–3.54 (PG-groups), 2.41–2.34 (d, broad, -N-(CH_3_)_2_), 1.41–1.35 (d, broad, -CH_3_); ^13^C-NMR: (CD_3_OD): δ (ppm) = 112.4 (C-CH_3_, ketal), 80.1–64.3 (PG-backbone), 48.6–43.3 (-N-CH_3_), 45.3–40.2 (-N-(CH_3_)_2_), 28.2 (C-CH_3_, ketal), 25.4 (C-CH_3_, ketal); IR (KBr) ῡ = 3142, 2975 (ketal; CH_3_), 2892, 2865, 1680, 1354, 1210 cm^−1^.

### 4.12. Procedure for the Synthesis of PG-cNH_2_ Containing 6-((tert-butoxycarbonyl(pyridin-2-ylmethyl)-amino)methyl)nicotinic Acid ***11***


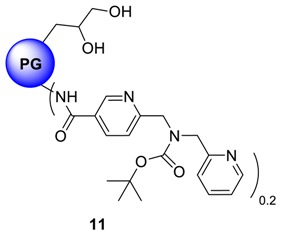


6-((*tert*-Butoxycarbonyl(pyridin-2-ylmethyl)amino)methyl)nicotinic acid (NF 135) (0.029 g, 0.087 mmol, 1.0 eq.) was dissolved in 3 mL p.a. DMF in a one necked 10 mL round-bottomed flask. To the resulting solution, EDCI (0.018 g, 0.096 mmol, 1.1 eq.) and catalytic amount of DMAP (0.001 mmol, 1 mol%) was added while stirring at 0 °C. After 15 min, a solution of PG-cNH_2_ (0.1 g, 0.43 mmol NH_2_ group, 20% of which will be functionalized with nicotinic acid moiety) in 1 mL p.a. DMF was added drop-wise to the reaction mixture for 30 min. The reaction was allowed to run for 24 h and the temperature was allowed to rise from 0 °C to r.t. The solvent was evaporated and the product was dialyzed in MeOH for 24 h. Yield: 72%; Conversion: quant.; ^1^H-NMR (500 MHz, CD_3_OD): δ (ppm) = 8.41 (1H, aromatic), 7.92–7.70 (3H, aromatic), 7.23–7.51 (3H, aromatic), 4.72–4.51 (functionalized primary PG-groups), 4.20–3.43 (PG-backbone), 2.53–2.22 (4H, -CH_2_-), 1.37 (3H, CH_3_) ^13^C-NMR: (CD_3_OD): δ (ppm) = 169.2, 150.4, 148.6, 131.8, 119.6, 128.6, 75.1, 29.3; IR (KBr) ῡ = 3564, 3121, 3002, 1745, 1645, 1599, 1409, 1326, 1089 cm^−1^.

### 4.13. Procedure for the Synthesis of PG-cNH_2_ Containing 6-(((2-(tert-butoxycarbonyl(pyridin-2-ylmethyl)-amino)ethyl)(propyl)amino)methyl)nicotinic Acid ***12***


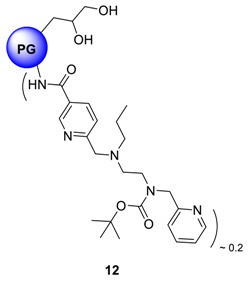


6-(((2-(*tert*-Butoxycarbonyl(pyridin-2-ylmethyl)amino)ethyl)(propyl)amino)methyl)nicotinic acid (KR 455), (0.050 g, 0.117 mmol, 1.0 eq.) was dissolved in 3 mL p.a. DMF in a one necked 10 mL round-bottomed flask. To the resulting solution, EDCI (0.025 g, 0.128 mmol, 1.1 eq.) and catalytic amount of DMAP (0.001 mmol, 1 mol%) was added while stirring at 0 °C. After 15 min, a solution of PG-cNH_2_ (0.027 g, 0.117 mmol NH_2_ group) in 1 mL p.a. DMF was added drop-wise to the reaction mixture for 30 min. The reaction was allowed to run for 24 h and the temperature was allowed to rise from 0 °C to r.t. The solvent was evaporated and the product was dialyzed in MeOH for 24 h. Yield: 76%; Conversion: quant.; ^1^H-NMR (500 MHz, CD_3_OD): δ (ppm) = 8.77 (bs, 1H, aromatic), 8.43–8.11 (bs, 3H, aromatic), 7.78–7.62 (bs, 4H, aromatic), 7.12–6.91 (bs, 3H, aromatic), 4.51 (-CH_2_-NHCO-), 4.01–3.34 (PG-backbone), 2.45–2.15 (-CH_2_-NCH_2_-CH_2_-), 1.41–1.45 (bs, -CH_2_ + CH_3_-, BOC), 0.84 (-CH_3_) ^13^C-NMR: (CD_3_OD): δ (ppm) = 167.1, 157.4, 153.5, 139.6, 127.8, 124.3, 79.4, 25.6; IR (KBr) ῡ = 3363, 3065, 3027, 2878, 1698, 1543, 1435, 1276, 1121, 855 cm^−1^. [An equivalent of 25% of all PG amino groups in the core were targeted, the functionalization ended up at ~20% after the reaction].

### 4.14. Procedure for the Synthesis of Maltose Modified PG Amine ***14***


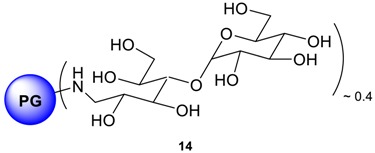


PG amine (0.25 g, 3.419 mmol NH_2_ group), D-(+)-maltose monohydrate (12.319 g, 34.189 mmol, 10.0 eq.), and borane-pyridine complex (4.30 mL, 3.177 g, 34.189 mmol, 10 eq., 8 M solution) were dissolved in sodium borate buffer (40.0 mL, 0.1 M) solution. The reaction solution was stirred at 50 °C for 7 d. The crude product was transferred directly to dialysis tube and purified by dialysis towards deionized water for 3 d. Maltose modified PG amine was obtained after freeze drying. Yield: 65% Conversion: 48% of all glycidol units based on anomeric proton at 5.1 ppm. ^1^H-NMR (500 MHz, D_2_O): δ (ppm) = 5.10–5.33 (bs, 1H, Sugar), 4.42–2.56 (PG-backbone + Sugar); ^13^C-NMR: (D_2_O): δ (ppm) = 100.4 (C-1), 81.7 (C-4), 75.8–69.3 (PG-backbone + C-maltose), 62.7–62.2 (6-C), 60.3 (6-C).

### 4.15. Procedure for the Synthesis of N-Acetylglucosamine Modified PG Amine ***15***


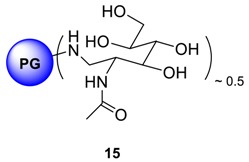


PG amine (0.25 g, 3.419 mmol NH_2_ group), *N*-acetylglucosamine (12.319 g, 34.189 mmol, 10.0 eq.), and borane-pyridine complex (4.30 mL, 3.177 g, 34.189 mmol, 10 eq., 8 M solution) were dissolved in sodium borate buffer (40.0 mL, 0.1 M) solution. The reaction solution was stirred at 50 °C for 7 days. The crude yellow colored solution was transferred directly to dialysis tube and purified by dialysis towards acidified water for 3 days. *N*-acetylglucosamine modified PG amine was obtained after freeze drying. Yield: 58%; Conversion 54% of all glycidol units; ^1^H-NMR (500 MHz, D_2_O): δ (ppm) = 4.51–3.23 (PG-backbone + hexose unit); 2.23–1.85 (bs, -CH_3_-); ^13^C-NMR: (D_2_O): δ (ppm) = 168.7 (C = O), 74.7–60.8 (PG-groups + Sugar unit), 58.7 (C-5), 53.2 (6-Ć), 48.7 (-CH_2_NH-), 21.9 (-CH_3_).

### 4.16. Procedure for the Synthesis of Polyglycerolamine Containing N-α-BOC-Histidine ***16***


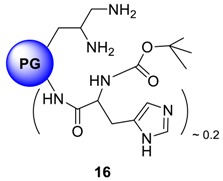


*N*-α-BOC histidine (0.035 g, 0.136 mmol, 1.0 eq.) was dissolved in 2 mL p.a. DMF in a one necked 10 mL round-bottomed flask. To the resulting solution, EDCI (0.029 g, 0.150 mmol, 1.1 eq.) and catalytic amount of DMAP (0.001 mmol, 1 mol%) was added while stirring at 0 °C. After 15 min, a solution of PG amine (0.1 g, 0.68 mmol NH_2_ group, 20% of which will be functionalized with histidine moiety) in 2 mL p.a DMF:water (3:1) was added drop-wise to the reaction mixture for 30 min. The reaction was allowed to run for 24 h and the temperature was allowed to rise from 0 °C to r.t. The solvent was evaporated and the product was dialyzed in MeOH for 24 h. Yield: 70%; Conversion: quant. ^1^H-NMR (500 MHz, CD_3_OD): δ (ppm) = 7.62 (bs, 1H, imidazole ring), 6.91 (bs, 1H, imidazole ring), 4.42–3.97 (functionalized PG-groups), 3.95–3.41 (PG-backbone), 3.07 (bs, 1H, -CH-), 2.88 (2H, -CH_2_-), 1.49 (bs, 3H, -CH_3_); ^13^C-NMR: (CD_3_OD): δ (ppm) = 160.8, 154.6, 143.5, 129.8, 124.3, 29.7; IR (KBr) ῡ = 2982, 2769, 1745, 1612, 1587,1523, 1434, 1369, 1275 cm^−1^.

## Figures and Tables

**Table 1:** Library of hPG-based Cu-complexing nanocarriers. * Compound **3f** indicates TMEDA bearing hPG systems at full functionalization level. Synthesis and characterization of compound **3f** (at full functionalization level) has been reported earlier [13]. Different partial functionalization of hPG, wherever used in this study, has been specified accordingly.

Compoundno.	Compound Code	Structure
		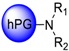
**3a**	PG_10_-DMA	R_1_ = R_2_ = -CH_3_
**3b**	PG_10_-DEA	R_1_ = R_2_ = -CH_2_-CH_3_
**3c**	PG_10_-DPA	R_1_ = R_2_ = -CH_2_-CH_2_-CH_3_
**3d**	PG_10_-DIPA	R_1_ = R_2_ = -CH(CH_3_)_2_
**3e**	PG_10_-DBA	R_1_ = R_2_ = -CH_2_-CH_2_-CH_2_-CH_3_
3f *	PG10-(TMEDA)_x_	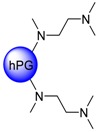
		X = Degree of functionalization, for 3f, X = 1.0
**8**	PG_m_-cNH_2_	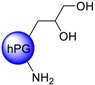
**8a**	m = 10 kDa	
**8b**	m = 5 kDa	
**10**	PG_10_-cTMEDA	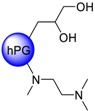
**11**	PG_10_-NF135	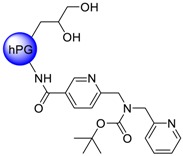
**12**	PG_10_-KR455	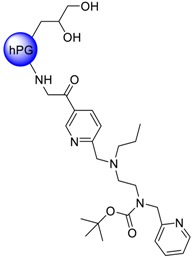
**14**	PG_10_-Mlt	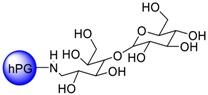
**15**	PG_10_-GLNC	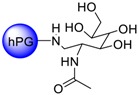
**16**	PG_10_-His	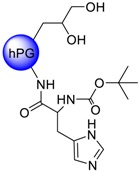

**Table 2:** Designing dialkylamine terminated PG. Range of amine groups and yields. Compound **3f** has not been included, as chemical data of the molecule has been published earlier [13].

Compound	R^1^ = R^2^	Yield (%)
**3a**	CH_3_-	60
**3b**	CH_3_CH_2_-	86
**3c**	CH_3_CH_2_CH_2_-	76
**3d**	(CH_3_)_2_-CH-	56
**3e**	CH_3_CH_2_CH_2_CH_2_-	68

**Table 3:** Cu^2+^ complexing stoichiometry as determined by ITC and UV-Vis spectroscopy.

Compound No.	Nanocarriers	Mole of Cu^2+^/mol of Nanotransporter	
		ITC	UV-Vis
3f (50%) *	PG_10_-TMEDA_0.5_	31	36
3f (10%) *	PG_10_-TMEDA_0.1_	14	15
**8a**	PG_10_-cNH_2_	26	31
**8b**	PG_5_-cNH_2_	10	17
**11**	PG_10_-NF 135	45	40
**12**	PG_10_-KR 455	55	48
**14**	PG_10_-Mlt	N/D	40
**15**	PG_10_-GLNC	N/D	42
**16**	PG_10_-His	35	N/D

N/D: not determined; It was not possible to accurately determine the saturation stoichiometry of Cu^2+^ by PG10-Mlt or PG10-GLNC system by ITC (inadequate model fitting), and PG_10_-His by UV-Vis spectroscopic techniques (very week LMCT signal). * % value inside parenthesis indicates the degree of functionalization of total number of hydroxyl groups/mol of hPG.

**Table 4:** Zeta potential of PG based Cu-encapsulating systems.

Compound No.	Nanocarriers	Zeta Potential (mV)
**11**	PG_10_-NF 135	(+) 36.0 ± 1.0
**12**	PG_10_-KR 455	(+) 38.5 ± 0.5
**3a**	PG_10_-DMA	(+) 28.0 ± 0.5
**14**	PG_10_-Mlt	(−) 1.5 ± 1.0
**15**	PG_10_-GLNC	(+) 5.0 ± 0.5
**16**	PG_10_-His	(+) 8.5 ± 1.0
